# Increased proportions of circulating PD-1^+^ CD4^+^ memory T cells and PD-1^+^ regulatory T cells associate with good response to prednisone in pulmonary sarcoidosis

**DOI:** 10.1186/s12931-024-02833-y

**Published:** 2024-05-07

**Authors:** Jelle R. Miedema, Lieke J. de Jong, Vivienne Kahlmann, Ingrid M. Bergen, Caroline E. Broos, Marlies S. Wijsenbeek, Rudi W. Hendriks, Odilia B. J. Corneth

**Affiliations:** Department of Pulmonary Medicine, Erasmus MC, University Medical Center, Doctor Molewaterplein 40, Rotterdam, 3015 GD The Netherlands

**Keywords:** Sarcoidosis, T cells, PD-1, CD25, Treatment, Prednisone

## Abstract

**Background:**

The treatment response to corticosteroids in patients with sarcoidosis is highly variable. CD4^+^ T cells are central in sarcoid pathogenesis and their phenotype in peripheral blood (PB) associates with disease course. We hypothesized that the phenotype of circulating T cells in patients with sarcoidosis may correlate with the response to prednisone treatment. Therefore, we aimed to correlate frequencies and phenotypes of circulating T cells at baseline with the pulmonary function response at 3 and 12 months during prednisone treatment in patients with pulmonary sarcoidosis.

**Methods:**

We used multi-color flow cytometry to quantify activation marker expression on PB T cell populations in 22 treatment-naïve patients and 21 healthy controls (HCs). Pulmonary function tests at baseline, 3 and 12 months were used to measure treatment effect.

**Results:**

Patients with sarcoidosis showed an absolute forced vital capacity (FVC) increase of 14.2% predicted (± 10.6, *p* < 0.0001) between baseline and 3 months. Good response to prednisone (defined as absolute FVC increase of ≥ 10% predicted) was observed in 12 patients. CD4^+^ memory T cells and regulatory T cells from patients with sarcoidosis displayed an aberrant phenotype at baseline, compared to HCs. Good responders at 3 months had significantly increased baseline proportions of PD-1^+^CD4^+^ memory T cells and PD-1^+^ regulatory T cells, compared to poor responders and HCs. Moreover, decreased fractions of CD25^+^ cells and increased fractions of PD-1^+^ cells within the CD4^+^ memory T cell population correlated with ≥ 10% FVC increase at 12 months. During treatment, the aberrantly activated phenotype of memory and regulatory T cells reversed.

**Conclusions:**

Increased proportions of circulating PD-1^+^CD4^+^ memory T cells and PD-1^+^ regulatory T cells and decreased proportions of CD25^+^CD4^+^ memory T cells associate with good FVC response to prednisone in pulmonary sarcoidosis, representing promising new blood biomarkers for prednisone efficacy.

**Trial registration:**

NL44805.078.13

**Supplementary Information:**

The online version contains supplementary material available at 10.1186/s12931-024-02833-y.

## Background

Sarcoidosis is a granulomatous multi-organ disease with variable disease course. The majority of patients presents with pulmonary involvement [1]. The decision to initiate treatment for sarcoidosis is based on an individual assessment of dangerous or progressive organ involvement and quality of life [2]. Glucocorticoids are recommended as first line choice [2]. However, the response to prednisone is highly variable and some patients do not respond to treatment [3]. Estimating the response to glucocorticoids can be challenging and prolonged prednisone use can have significant toxicity and side effects [2–4]. There is an unmet need to develop biomarkers that predict therapeutic response [5].

Granuloma formation in sarcoidosis results from an exaggerated immune response to an unidentified antigen in genetically susceptible individuals. Several disease-triggering antigens have been suggested that may initiate the inflammatory cascade [6]. Convincing evidence has been obtained that cluster of differentiation (CD)4^+^ T cell immunology is central to sarcoidosis disease course [6–8]. In bronchoalveolar lavage fluid (BALF) and mediastinal lymph nodes (MLN) of patients with sarcoidosis, the frequencies of IL-17^+^ or IL-17^+^IFNγ^+^ memory T cells are increased [7–9]. Moreover, aberrant activation of T helper (Th)17 and regulatory T (Treg) cells was found, characterized by decreased expression of co-inhibitory receptor cytotoxic T-lymphocyte antigen 4 (CTLA4) in BALF and MLN [10]. Additionally, increased proportions of circulating CD4^+^ T cells with expression of checkpoint molecule programmed death-1 (PD-1) and an exhausted phenotype were found in progressive sarcoidosis relative to patients with disease resolution and healthy controls (HCs) [11]. Proportions of circulating PD-1^+^CD4 central- and effector memory T cells in sarcoidosis were increased and these cells showed decreased proliferative capacity [12]. During resolution of sarcoidosis, PD1^+^CD4^+^ T cells in peripheral blood (PB) decreased and normalization of immune function was observed, indicating that the phenotypical and functional T cell changes can be reversible [11]. Interestingly, cancer treatment with anti-PD-1 or CTLA4-immune checkpoint blockade can cause a drug-induced sarcoidosis-like disease, further highlighting the link between aberrant T cell activation and sarcoidosis [13].

Early CD4^+^ T cell activation leads to upregulation of the interleukin-2 receptor (IL-2R)/CD25 [14]. Subsequently, CD25 is released into the circulation as soluble IL-2R (sIL2R), which can be used to assess sarcoidosis disease activity [15]. It correlates with proportions of CD4^+^ T cells in the BALF and acute disease [16–18]. However, multiple studies failed to demonstrate a correlation between sIL2R and baseline pulmonary function, disease progression or response to glucocorticoid treatment [16, 19, 20]. Thus, despite strong evidence for critical involvement of T cells in sarcoidosis, it remains unknown whether the phenotype of circulating T cells can be used as therapeutic biomarker in sarcoidosis.

In this study, we hypothesized that the PB CD4^+^ T cell phenotype of patients with sarcoidosis might correlate with response to prednisone treatment. We evaluated frequencies and phenotypes of PB T cell populations in newly diagnosed patients with pulmonary sarcoidosis before prednisone treatment started, as well as in HCs. We correlated these parameters with lung function response and we investigated the effect of prednisone treatment on circulating T cells of patients.

## Methods

### Study design and subjects

In this prospective multicenter study, treatment-naïve patients with a diagnosis of pulmonary sarcoidosis according to the ATS/ERS/WASOG criteria were included [21]. Ethics approval was obtained from the Erasmus Medical Center (MEC-2013-244). All patients and healthy control (HC) subjects provided written informed consent. Previously, we reported on short-term response in hospital and home-based spirometry during prednisone treatment in this cohort (Trial Registration: NL44805.078.13) [22].

All patients had a baseline forced vital capacity (FVC) < 85% and parenchymal lung abnormalities. Prednisone treatment was standardized for the first 3 months: 4 weeks 40 mg (mg)/day, 2 weeks 30 mg/day, 2 weeks 20 mg/day, 2 weeks 15 mg/day, 2 weeks 10 mg/day. Hereafter, a maintenance dose of 5–10 mg/day was continued up until 1 year. At any time, the treating physician could decide to deviate from this treatment schedule if the clinical situation demanded so. In-hospital pulmonary function measurements of FVC and diffusion capacity for carbon monoxide (DLCO) were conducted at baseline, month 1, 3, 6, 9 and 12 and peripheral blood for immunological analysis was sampled at baseline, month 3 and 12. We defined good initial lung function responders at 3 months as patients with an absolute FVC predicted increase of ≥ 10% and poor responders as patients with < 10% FVC predicted increase during treatment, based on previously reported lung function response in sarcoidosis [22].

### Sample processing and flow cytometry

Blood samples were collected in EDTA tubes and PB mononuclear cells (PBMCs) were obtained by ficoll density separation and stored at -180 °C in RPMI medium containing 10% fetal calf serum and 10% dimethyl sulphoxide, according to standard procedures. Flow cytometric analyses were performed as previously described [23]. Antibodies used for intra- and extracellular staining are listed in Supplementary Table 1.

Flow cytometry standard (FCS) files obtained with the BD FACSymphonyTM A5 Cell analyzer were preprocessed and quality control was performed using peak extraction and cleaning oriented quality control (PeacoQC) [24]. Files were first preprocessed using Flowcore Package followed by peak detection and outlier removal using PeacoQC package in RStudio (v4.1.2). Clean FCS files were analyzed using FlowJo v10 (Tree Star Inc Software). Samples that contained > 70% debris or passed the PeacoQC quality control but > 50% of events had to be removed because of poor quality, were excluded. Percentage or geometric mean fluorescent intensity (gMFI) of activation markers were only calculated from gated fractions containing > 100 events.

### Statistical analysis

Statistical analyses were performed using GraphPad Prism 9 software (GraphPad Software Inc; San Diego, CA, USA), which was also used to design graphs and to calculate statistics on flow cytometric analyses. The comparison of means of continued variables measured at different time points were tested with the paired student t-test. A Mann-Whitney U test was used to calculate significant differences between two unpaired groups. Wilcoxon matched-pairs signed rank test was used for paired samples. In case of multiple group comparisons, statistical analyses were performed by Kruskal-Wallis test combined with a Dunn’s multiple comparison test. Spearman correlation was used to calculate significance of the strength of linear correlations between paired data represented in a scatterplot. *P* values < 0.05 were considered significant. In the figures, significant *p* values are displayed as *p < 0.05, **p < 0.01 and ***p < 0.001.

## Results

In this study 25 patients with pulmonary sarcoidosis were enrolled, three patients were excluded from our analyses. One patient had been using immunosuppressive therapy, one patient was not able to perform a technical correct pulmonary function test and one patient did not have paired blood samples available. No patients with Löfgren’s syndrome were included. The baseline demographic and clinical characteristics are shown in Table 1. Additionally, 21 age- and sex-matched HC subjects were included for comparison.

**Table 1:** Baseline characteristics of participating subjects

	**Control**	**Pulmonary sarcoidosis**
**Subjects**	*N* = 21	*N* = 22
**Age** (mean, ± SD)	47.1 (± 9.7)	43.9 (± 10.2)
**Female/male**	10(48) / 11(52)	8(36) / 14(64)
**Ethnicity**		
Black / white / other		6(27)/12(55)/ 4(18)
**Smoking**		
No/yes/former		15(68)/ 2(9)/ 5(23)
**Diagnosis confirmed by**		
Clinical- radiological features and granuloma’s in tissue biopsy n, (%)		18 (82)
Clinical- radiological features and BALF CD4/CD8 ratio > 3.5 n, (%)		3 (14)
Clinical- radiological features n, (%)		1 (4)
**Pulmonary function**		
Forced vital capacity (FVC) in % predicted (mean, ± SD)		70.6 (± 13.3)
Diffusion capacity (DLCOc) in % predicted (mean, ± SD)		62.5 (± 19.0)
**Scadding stage chest X-ray,** n, (%)		
0		1 (5)^a^
1		1 (5)
2		15 (68)
3		4 (18)
4		0
Unknown		1 (5)
**Extra thoracic involvement,** n, (%)		
Skin		3 (14)
Eyes		3 (14)
Liver		3 (14)
Spleen		2 (9)
Central nervous system		0
Cardiac		0
**Biomarkers**		
ACE (*n* = 21) mmol/L (median, IQR)		75 (13–284)
Soluble interleukin receptor (sIL2R; *n* = 15) U/L (median, IQR)		9423 (3000–30400)

^a^Confirmed pulmonary parenchymal involvement on chest computed tomography (CT)

### Patients with sarcoidosis show variable increase in FVC during prednisone treatment

**Fig. 1:**
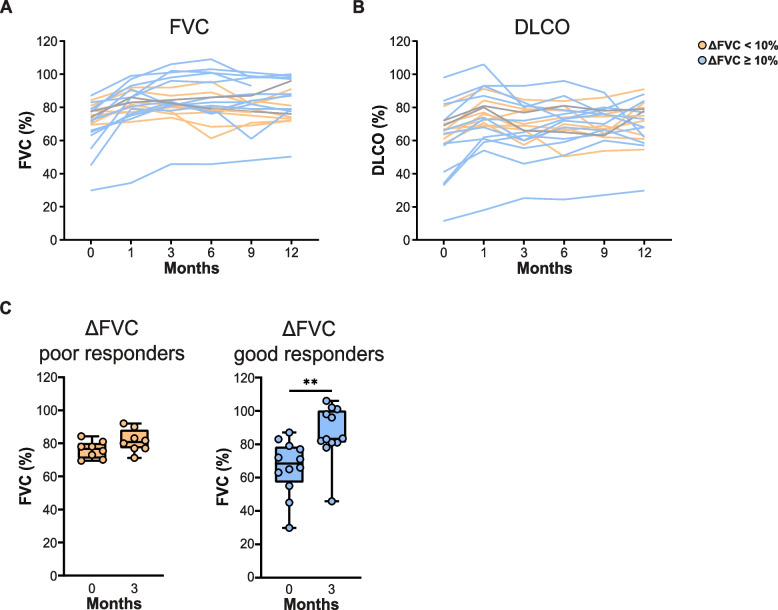
Pulmonary function of patients with sarcoidosis during 12 months of treatment. **A**, **B** In hospital measurement of Forced Vital Capacity (FVC) in % predicted (**A**) and diffusion capacity of the lungs for carbon monoxide corrected for haemoglobin (DLCOc) in % predicted (**B**). Each line represents one patient. Blue lines represent patients with ≥ 10% absolute FVC increase between 0 and 3 months. Orange lines represent patients with < 10% FVC increase in 3 months. Two patients had missing FVC data at the 3 months measurement (grey lines). **C** Boxplots indicating measured FVC in % predicted before and after 3 months of prednisone in two treatment outcome groups. Depicted are poor responders, defined as patients with < 10% absolute FVC increase between 0 and 3 months (*left*) and good responders, defined as patients with ≥ 10% absolute FVC increase between 0 and 3 months (*right*). Wilcoxon matched-pairs signed rank test was used to calculate significant differences between 0 and 3 months and 0 and 12 months. Mann-Whitney U test was used to calculate significant differences between two groups

During the first 3 months, all patients were treated according to the predefined prednisone treatment schedule outlined in the Methods section. At 12 months, 15 patients used prednisone ≤ 10 mg, one patient had missing data, one patient used 15 mg prednisone daily and five patients were not using prednisone anymore. Glucocorticoid-sparing therapy with methotrexate was initiated in five patients and azathioprine in one patient. During treatment with prednisone, FVC improved significantly with a mean increase of 14.2% (± 10.6, *p* < 0.0001) between baseline and 3 months (*n* = 20), and 13.5% (± 12.6, *p* < 0.0001) between baseline and 12 months (*n* = 22) (Fig. 1A). The DLCO did not change significantly between baseline and 3 months, but we observed a mean increase of 9.5% (± 16.8, *p* = 0.01) between baseline and 12 months (Fig. 1B). These data show that the increase in FVC during the first 3 months had a wide range. Using the ≥ 10% FVC predicted cut-off, 12 patients had a good treatment response with a significant median FVC increase of 14.8% (*p* = 0.0024) while the remaining eight patients were poor responders and had a non-significant median FVC increase of 4.2% (*p* = 0.1518) at 3 months follow up compared with baseline (Fig. 1C). Of the good responders at 3 months, 10 (83.3%) also had a good FVC response at 12 months, while 1 patient (8.3%) had a poor response and 1 patient (8.3%) had missing FVC data at 12 months. There was no significant difference in baseline FVC or DLCO between patients with good and poor FVC response at 3 months.

###  In patients with sarcoidosis, circulating CD4^+^ memory and regulatory T cells display an aberrantly activated phenotype

Using flow cytometry, we measured the proportions of PB T cell subsets of patients with sarcoidosis and HCs. The gating strategy is outlined in Suppl. Figure 1. Tregs were gated as CD127^−^CD25^high^CD4^+^ T cells (which were high in FoxP3 expression, Suppl.Fig. 1B). The populations of CD127^+^CD25^low^CD4^+^ T cells were divided into CD27^+^CD45RA^+^CD45RO^−^ naïve T cells (Tn) and CD45RA^−^CD45RO^+^ memory T cell (Tm) fractions. Proportions of CD4^+^, CD8^+^ T cells and γδ T cells (of CD3^+^ T cells) were unaltered in patients (Suppl. Figure 2AB). Within the CD4^+^ T cell subset, proportions of Tn and Tm cells were also unaltered. In line with previous results [25], increased proportions of Tregs were found in sarcoidosis compared with HCs (Suppl. Figure 2C).

We subsequently evaluated the expression of multiple surface markers that are known to be upregulated upon T cell activation (Suppl. Table 1). Activation marker expression on the surface of CD8^+^ T cells and CD4^+^ Tn cells was not significantly different between patients and HCs or across treatment response groups. In contrast, within the fraction of circulating CD4^+^ Tm cells, we found decreased proportions of CD25^+^ and CD28^+^ Tm cells in sarcoidosis compared with HCs, only reaching significance for CD25^+^ Tm cells (Suppl. Figure 3A). Hereby, the CD25^+^ CD4^+^ Tm cells showed high levels of CD127 expression, and could therefore readily be distinguished from CD25^hi^CD127^−^ Tregs, as shown in suppl. Figure 1.

Additionally, proportions of CTLA4^+^ and PD-1^+^CD4^+^ Tm were significantly increased in patients.

Compared with HCs, Tregs in patients with sarcoidosis displayed increased expression of CD25 and CD95/Fas (Suppl. Figure 3B). Whereas proportions of Tregs expressing CD28 were high and unaltered, the fractions of CTLA4^+^ and PD-1^+^Tregs were increased in sarcoidosis, compared with HCs.

Taken together, we conclude that in PB of treatment-naïve patients with sarcoidosis CD4^+^ Tm and Tregs displayed an aberrantly activated phenotype, compared with HCs.

###  Increased proportions of circulating PD-1^+^CD4^+^ memory T cells in sarcoidosis patients with good response to prednisone

**Fig. 2:**
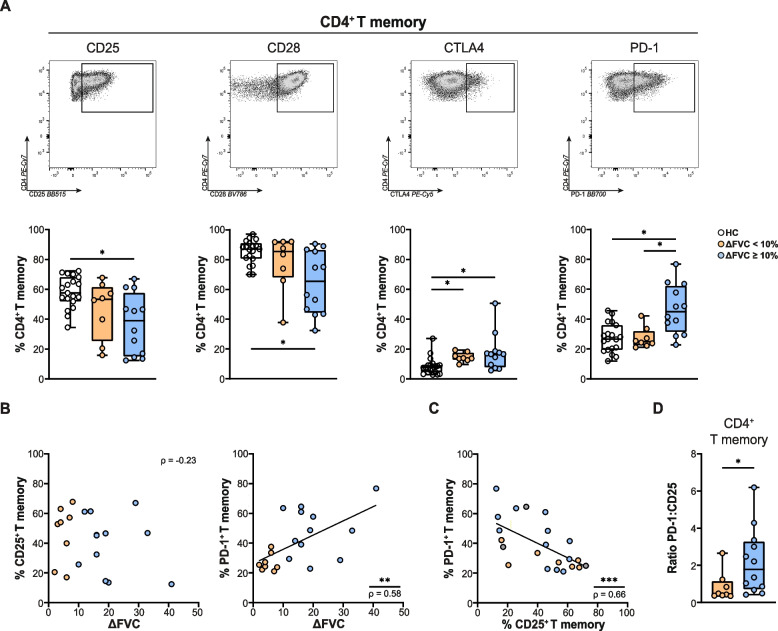
Activation marker expression on CD4^+^ memory T cells in sarcoidosis and correlation with prednisone response. **A** Representative flow cytometry dot plots (*top*) and proportions of CD4^+^ memory T cells positive for CD25, CD28, CTLA4 and PD-1 expression (*bottom*) in healthy controls (HCs), sarcoidosis patients with < 10% (poor response) and ≥ 10% (good response) absolute FVC % predicted increase in 3 months. **B** Scatter plots depicting correlation coefficients with *p*-value between absolute increase in FVC % predicted (∆ FVC) between baseline and 3 months and proportions of CD4^+^memory T cells (in %) positive for CD25 *(left)* or PD-1 *(right)*. **C** Scatter plot depicting correlation coefficient with *p*-value between proportions of CD4^+ ^memory T cells positive for CD25 (in %) and for PD-1. **D** Ratio of the proportions of CD4^+^ memory T cells positive for PD-1 over the proportions positive for CD25 in patients with poor and good response. Symbols represent individual values in HCs (open circles) and patients with sarcoidosis with ∆ FVC% predicted of < 10% (orange circles) and ∆ FVC% predicted of ≥ 10% (blue circles) between baseline and 3 months. All data were measured by flow cytometry. Mann-Whitney U test was used to calculate significant differences between two groups. In case of multiple group comparisons, statistical analysis was calculated using Kruskal-Wallis test combined with a Dunn’s multiple comparison. Spearman correlation was used to calculate significance of linear correlations between paired data in the scatterplots

Next, we investigated whether expression of surface markers on sarcoidosis CD4^+^ Tm cells at baseline correlated with the response to prednisone. Hereby, we compared patients with sarcoidosis and good response to prednisone to poor responders and HCs (Fig. 2A). While both treatment response groups had decreased proportions of circulating CD25^+^ and CD28^+^ Tm compared with HCs, this only reached significance in the good prednisone responders. Both outcome groups had increased proportions of CTLA4^+^ Tm cells compared to HCs. Interestingly, only good responders had significantly increased baseline proportions of PD-1^+^ Tm cells, compared to other patients with sarcoidosis and HCs (Fig. 2A). We found that the surface expression of PD-1 in the good responder group was higher than in the poor responder group in all T cell subsets analysed, including Th1, Th17 and Th17.1, as well as non-Th1/Th17 cells (based on CXCR3 and CCR6 expression).

We subsequently correlated for each patient the proportions of circulating CD25^+^ and PD-1^+^CD4^+^ Tm cells with the values for the absolute increase in FVC (% predicted) between baseline and 3 months. Whereas the FVC increase did not correlate with the proportions of CD25^+^ CD4^+^ Tm cells, we found a significant positive correlation between FVC increase and PD-1^+^CD4^+^ Tm cells (*r* = 0.58, *p* < 0.001) (Fig. 2B). Proportions of CD25^+^ and PD-1^+^CD4^+^ Tm cells showed a significant inverse correlation (*r* = -0.65 *p* < 0.001) (Fig. 2C). Hereby, the ratio of the proportions of PD-1^+^ over CD25^+^ CD4^+^ Tm cells was significantly higher in good responders than in poor responders (Fig. 2D).

In summary, increased baseline proportions of circulating PD-1^+^CD4^+^ Tm cells significantly associated with good FVC response to prednisone in patients with sarcoidosis.

###  Increased proportions of circulating PD-1^+^ regulatory T cells in patients with sarcoidosis and good response to prednisone

**Fig. 3:**
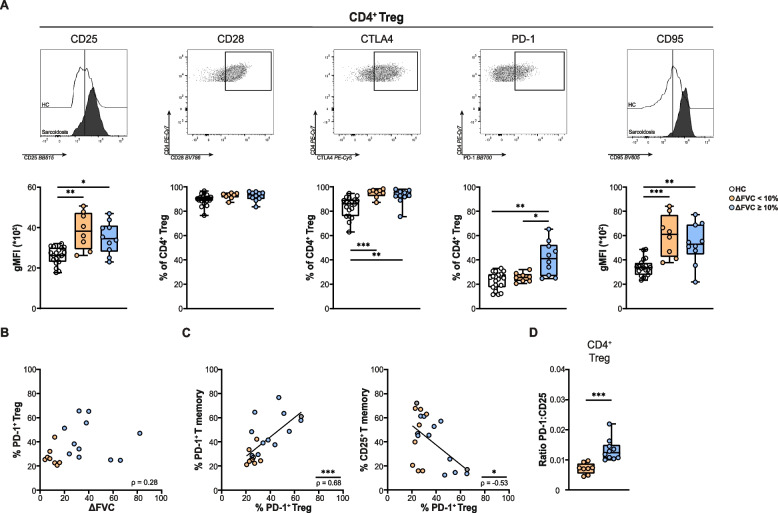
Activation marker expression on regulatory T cells in sarcoidosis and correlation with prednisone response. **A** Representative flow cytometry histograms of CD25 and CD95/Fas (gMFI) expression and dot plots of gated CD4^+^ regulatory T cells showing the expression of CD28, CTLA4 and PD-1 (*top*), and box plots showing the expression in healthy controls (HCs), sarcoidosis patients with < 10% and ≥ 10% increase in FVC % predicted (poor and good responders, respectively) in 3 months (*bottom*). **B** Scatter plot depicting correlation between FVC % predicted and proportions of cells within Tregs that are positive for PD-1, with ρ-value. **C** Scatter plot depicting correlation between proportions of Tregs positive for PD-1 and proportions of CD4^+^ memory T cells positive for PD-1 *(left)* or CD25 *(right)*. **D** Ratio of the proportions of Tregs expressing PD-1 (in %) over the CD25 expression level (gMFI) on Tregs patients with a poor and a good response (∆FVC of < 10% and ≥ 10% in 3 months, respectively). Symbols represent individual values in HCs (open circles) and sarcoidosis patients with ∆FVC of < 10% (orange circles) and ∆ FVC ≥ 10% (blue circles) between baseline and 3 months. All data were measured by flow cytometry. Mann-Whitney U test was used to calculate significant differences between two groups. In case of multiple group comparisons, statistical analysis was calculated using Kruskal-Wallis test combined with a Dunn’s multiple comparison. Spearman correlation was used to calculate significance of linear correlations between paired data in the scatterplots

We subsequently investigated the phenotype of circulating Tregs in the two treatment outcome groups and HCs. Tregs displayed increased expression of CD25 and CD95 in both sarcoidosis outcome groups compared to HCs. Proportions of CD28^+^ Tregs were high in all groups and not significantly different between patients and HCs (Fig. 3A). Proportions of CTLA4^+^ Tregs were increased in sarcoidosis compared with HCs. Similar to our findings for PD-1^+^CD4^+^ Tm cells, we found significantly increased proportions of PD-1^+^ Tregs in good responders compared with poor responders and HCs (Fig. 3A). No correlation was found between proportions of CD25^+^ or PD-1^+^ Tregs and absolute increase in FVC % predicted after 3 months (Shown for PD-1^+^ Tregs in Fig. 3B).

Within sarcoidosis patients, the proportions of PD-1^+^ Tregs showed a positive correlation with the proportions of PD-1^+^CD4^+^ Tm cells (*r* = 0.68 *p* < 0.001), and an inverse correlation with CD25^+^CD4^+^ Tm cells (*r* = -0.53 *p* < 0.05) (Fig. 3C). Interestingly, the PD-1^+^ to CD25^+^ ratio in Tregs markedly separated patients with good glucocorticoid response from patients with poor response (*p* < 0.001) (Fig. 3D).

We also tested the correlation between the levels of sIL2R in the serum of patients (*n* = 15) and the FVC response during 3 months of prednisone treatment. In line with published data indicating poor sIL2R biomarker performance [16], we found no significant correlation (Suppl. Figure 4A). Additionally, sIL2R did not significantly correlate with baseline proportions PD-1^+^ or CD25^+^ Tm and Tregs (Suppl. Figure 4B).

Collectively, these results indicate that PB Tregs in sarcoidosis have an activated phenotype. The proportions of PD-1^+^ Tregs at baseline correlated with good FVC response at 3 months of prednisone treatment.

###  Prednisone treatment reverses the aberrantly activated phenotype of circulating CD4^+^ memory T cells and regulatory T cells in sarcoidosis

**Fig. 4:**
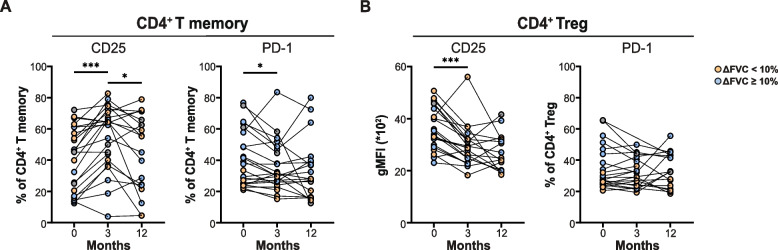
Proportions of CD4^+^PD-1^+^ and CD25^+ ^memory and regulatory T cells in patients with sarcoidosis during treatment. **A** Paired measurements of proportions of CD4^+^ memory T cells positive for CD25 (*left*) and PD-1 (*right*) at baseline, 3 and 12 months. **B** Paired measurement of gMFI values for CD25 (*left*) and the proportions of PD-1-expressing cells in the Treg population at baseline, 3 and 12 months. Each connecting line represents one patient. Symbols represent individual values of patients with sarcoidosis in the two treatment outcome groups with ∆ FVC% predicted of < 10% (poor response, orange circles) and ∆ FVC% predicted of ≥ 10% (good response, blue circles) in 3 months. All data were measured by flow cytometry. Wilcoxon matched-pairs signed rank test was used to calculate significant differences between 0 and 3 months, 3 and 12 months and 0 and 12 months

To elucidate treatment effects on PD-1 and CD25 surface expression on CD4^+^ Tm cells and Tregs in sarcoidosis, we compared baseline blood samples with paired samples at 3 and 12 months. During the first 3 months of prednisone treatment (which was standardized, see Methods), proportions of CD25^+^ CD4^+^ Tm cells significantly increased, while proportions of PD-1^+^CD4^+^ Tm cells significantly decreased, compared with baseline values (Fig. 4A). Also, proportions of CD25^+^ and PD-1^+^ Tregs decreased, but this only reached significance for CD25^+^ Tregs (Fig. 4B). At 12 months of treatment (in which prednisone was differentially tapered across patients, see Methods), proportions of Tm cells and Tregs expressing either CD25 or PD-1 were not different from baseline.

Together, these findings indicate that high dose prednisone treatment reversed the aberrant baseline expression of CD25 and PD-1 on CD4^+^ Tm and Treg, but this effect waned after tapering of prednisone at 12 months.

###  Baseline proportions of CD25^+^ and PD-1^+^CD4^+^ memory T cells predict good lung function response after 1 year

**Fig. 5:**
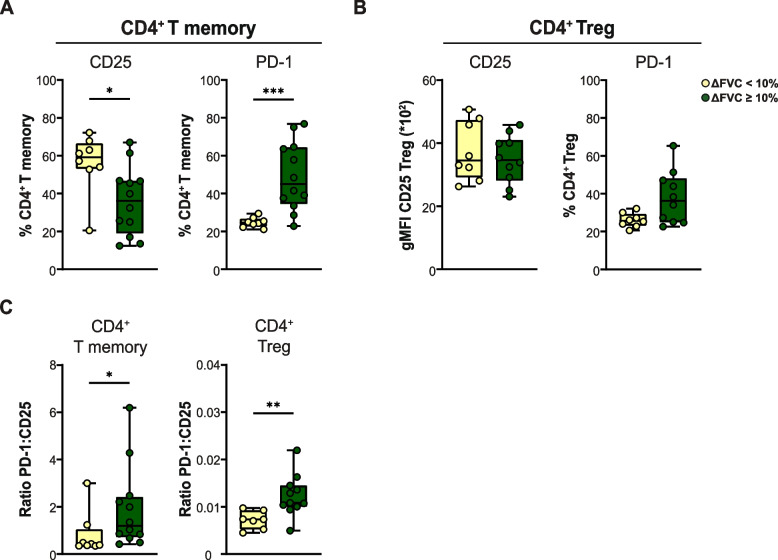
Baseline proportions of PD-1^+^ or CD25^+^ CD4^+^ memory and Treg and correlation with 12 month FVC response. **A** Baseline proportions of CD4^+^ memory T cells positive for CD25 (*left*) and PD-1 (*right*) in patients with sarcoidosis in the two treatment outcome groups defined by < 10% (poor response) and ≥ 10% (good response) FVC (% predicted) increase at 12 months compared to baseline. **B** Expression of CD25 on Tregs (gMFI) (*left*) and proportions of CD4^+^ regulatory T cells positive for PD-1 (*right*) in sarcoidosis treatment outcome groups with < 10% and ≥ 10% absolute FVC (% predicted) increase at 12 months compared to baseline. **C** Ratio of the proportion of CD4^+^ memory T cells expressing PD-1 over de proportions that express CD25 in the two response groups (*left*) and the ratio of the proportion of Tregs that are PD-1^+^ over the expression level of CD25 (in gMFI). Symbols represent individual values for patients in the indicated response groups. All data were measured by flow cytometry. Mann-Whitney U test was used to calculate significant differences between two groups

Finally, we investigated if the baseline proportions of CD25^+^ and PD-1^+^CD4^+^ Tm cells and Tregs correlated with good long-term treatment response, defined as ≥ 10% absolute FVC increase between baseline and 12 months. At 1 year follow up, there was no significant difference in steroid dose between the two treatment outcome groups. Good and poor responders used median 7.5 mg and 5.0 mg prednisone daily, respectively (*p* = 0.995). We found significantly decreased proportions of CD25^+^ and significantly increased proportions of PD-1^+^CD4^+^ Tm cells at baseline in patients with good FVC response, compared with poor responders at 12 months (Fig. 5A). We observed a trend of increased proportions of PD-1^+^ Tregs in patients with good FVC response compared with poor responders, but this did not reach significance (Fig. 5B). The ratio between PD-1^+^and CD25^+^ proportions for both Tm and Treg at baseline correlated significantly with good response to prednisone at 12 months compared with poor responders (Fig. 5C). No correlation between activation marker expression on T cell subsets or FVC and DLCOc between baseline and 12 months was found.

In summary, these data show that high baseline ratios of PD-1 to CD25 expression, both on CD4^+^ Tm cells and on Tregs correlated significantly with FVC response at 12 months.

## Discussion

Stratifying patients with a high likelihood to benefit from glucocorticoid treatment is essential to optimize personalized sarcoidosis care. Currently, no predictive biomarker is available and first-line prednisone is recommended for all patients with sarcoidosis with a treatment indication [2]. Here, we show for the first time significant correlations with the phenotype of circulating T cells at baseline and ≥ 10% absolute FVC increase during prednisone treatment in pulmonary sarcoidosis. In particular, increased proportions of PD-1^+^ cells and decreased proportions of CD25^+^ cells within the CD4^+^ Tm cell population, as well as increased fractions of PD-1^+^ cells in the Treg population, correlated with a good treatment response. Importantly, for Tm cells as well as Tregs the ratio of PD-1^+^ to CD25^+^ cells correlated with treatment response, both at 3 and at 12 months. These findings indicate the potential of PD-1^+^ and CD25^+^ expression levels on circulating T cell populations as new biomarkers to predict glucocorticoid therapy response in pulmonary sarcoidosis. In contrast, we did not detect an association with treatment outcome for serum sIL2R, a biomarker currently used in clinical care.

PD-1 is upregulated on all T cells following T-cell receptor-mediated activation and can remain elevated during persistent antigen presentation [26]. Increased expression of PD-1 negatively regulates T cell proliferation and effector function and contributes to peripheral tolerance and T cell exhaustion, as observed in tumor microenvironments [26]. In progressive sarcoidosis, circulating CD4^+^ T cells display an exhausted phenotype with high PD-1^+^ proportions [11]. We previously described comparable baseline proportions of circulating PD-1^+^ CD4^+^ Tm cells in patients with spontaneous disease resolution and progressive sarcoidosis [27]. This finding suggests the Tm phenotype itself does not contribute to disease resolution. In the current study, the remarkable PD-1^high^CD25^low^ phenotype of CD4^+^ Tm cell population in good responders may indicate a selection of patients with less successful antigen clearance due to aberrant T cell activation.

In line with previous work, we found an aberrantly activated phenotype of PB Tregs in patients with sarcoidosis, characterized by increased levels of CD25 and CD95 expression and increased proportions of CTLA4-expressing cells [25]. Considering Tregs, only increased proportions of PD-1^+^ cells associate with good treatment efficacy. Circulating PD-1^high^ Tregs have impaired suppressive function and signs of exhaustion, as shown in patients with malignant glioma [28]. In sarcoidosis, a reduced regulatory capacity and survival of Tregs was described previously, whereby restoration of their function was associated with disease resolution [25, 29]. Here, we demonstrate a correlation between proportions of PD-1^+^ Tregs and PD-1^+^CD4^+^ Tm cells. Mechanistically, it is conceivable that the impaired immunosuppressive function of exhausted PD-1^high^ Tregs contributes to ongoing activation of circulating CD4^+^ Tm cells. However, to elucidate the function and possible exhaustion of circulating PD-1^high^ Tregs in sarcoidosis, additional research is needed.

Given the lack of correlation between sIL2R levels and FVC response to prednisone in this study, overall T cell activation in sarcoidosis probably does not predict glucocorticoid efficacy. Prednisone has broad immunosuppressive effects on immune cells implicated in sarcoidosis pathogenesis [6, 30]. Importantly, it inhibits the activation and differentiation of effector T cells and supresses the production of pro-inflammatory cytokines involved in granulomatous inflammation [30]. In this study, prednisone treatment was associated with reversal of the aberrantly activated phenotype of PB memory and regulatory T cells in sarcoidosis. However, the exact mechanisms by which specifically PD-1^+^CD4^+^ Tm cells and PD-1^+^ Tregs predispose to good prednisone sensitivity in sarcoidosis need to be elucidated. Additional research is needed to explore whether the association is specific for sarcoid granulomatous inflammation or reflects general mechanisms that fuel glucocorticoid sensitivity. In cancer treatment, the balance of PD-1 expression between CD8^+^ effector T cells and regulatory T cells in the tumor microenvironment associates with the therapeutic effect of PD-1 blocking immunotherapy [31].

Our study has some limitations to address. First, our findings need to be validated in a larger patient cohort before clinical application is possible. Second, we were able to correlate T cell phenotype with FVC but not with the DLCO response. This may be explained by high DLCO variability in response to treatment and therefore it will be valuable to explore this in larger groups of patients. Third, the number of patients included in this study was limited and prospective follow-up was 1 year. We were therefore not able to correlate T cell phenotypes with clinically relevant disease relapse upon prednisone withdrawal, which is estimated to occur in 20–80% of patients after initial therapy [2, 4, 32].

## Conclusion

The current study shows increased baseline proportions of circulating PD-1^+^CD4^+^ memory and regulatory T cells in patients with sarcoidosis and good initial FVC response to prednisone. Additionally, increased PD-1^+^ and decreased CD25^+^ CD4^+^ memory T cell proportions associate with favorable long term FVC response. These findings reveal a promising blood biomarker for prednisone efficacy in treatment-naïve patients with pulmonary sarcoidosis.

### Supplementary Information

Supplementary Material 1: Supplementary Table 1. Antibodies used for intra- and extracellular staining.

Supplementary Material 2: Suppl. Figure 1. Gating strategy of human T cell subsets. Gating strategy for the indicated human T cell populations in peripheral blood mononuclear cell fractions. Resting T cells: CD127^+/-^CD25^-^-, Activated T cells: CD127^+^ CD25^+^, Tregs: CD127^-^ CD25^high^.

Supplementary Material 3: Suppl. Figure 2. Proportions of T cell subsets in healthy controls and patients with sarcoidosis. (A) Proportions of CD3^+^ T cells of total lymphocytes in peripheral blood mononuclear cell fractions. (B) Proportions of CD4^+^, CD8^+^ and γδ^+^ T cells of total CD3^+^ T cells (C) Proportions of CD4^+^ T naïve, T memory and Treg cells of total CD4^+^ T cells. Symbols represent individual values in healthy controls (HCs; open circles) and total sarcoidosis patients (Sarcoidosis; blue circles). All data were measured by flow cytometry. Mann-Whitney U test was used to calculate significant differences between two groups. *p < 0.05, **p < 0.01 and ***p < 0.001.

Supplementary Material 4: Suppl. Figure 3. Expression of activation markers on CD4^+^ memory T cells and Tregs in healthy controls and patients with sarcoidosis. (A) Proportions of CD4^+^ memory T cells expressing CD25, CD28, CTLA4 and PD-1. (B) Proportions of Tregs expressing CD28, CTLA4 and PD-1. Expression of CD25 and CD95 on Tregs is depicted in gMFI. Symbols represent individual values in healthy controls (HCs; open circles) and total sarcoidosis patients (Sarcoidosis; blue circles). All data were measured by flow cytometry. Mann-Whitney U test was used to calculate significant differences between two groups. *p < 0.05, **p < 0.01 and ***p < 0.001.

Supplementary Material 5: Suppl. Figure 4. Serum concentration of soluble IL-2 receptor does not correlate with FVC response or T cell activation marker expression. (A) Scatter plots depicting correlation coefficients with *p*-value between absolute increase in FVC % predicted (∆ FVC) between baseline and 3 months and serum concentration of soluble IL-2 receptor (sIL2R) in U/L in sarcoidosis patients with < 10% and ≥ 10% absolute FVC % predicted increase in 3 months (B) Scatter plot depicting correlation coefficient between proportions of CD4^+ ^memory T cells positive for CD25 (in %; *left)* and for PD-1 (*right)* and serum concentration of soluble IL-2 receptor (sIL2R) in U/L in sarcoidosis patients with < 10% and ≥ 10% absolute FVC % predicted increase in 3 months (C) Scatter plot depicting correlation coefficient between baseline expression level of CD25 (in gMFI) (*left*) or the proportions of Tregs that express PD-1 (*right*) and serum concentration of soluble IL-2 receptor (sIL2R) in U/L in sarcoidosis patients with < 10% and ≥ 10% absolute FVC % predicted increase in 3 months. Symbols represent individual values in patients with sarcoidosis in the two response groups, as indicated.

## Data Availability

No datasets were generated or analysed during the current study.

## Abbreviations

BALF: Bronchoalveolar lavage fluid
CD: Cluster of differentiation
CTLA4: Cytotoxic T-lymphocyte antigen 4
DLCO: Diffusion capacity for carbon monoxide
FVC: Forced vital capacity
HCs: Healthy controls
MLN: Mediastinal lymph node
PB: Peripheral blood
PD-1: Programmed death-1
sIL2R: Soluble IL-2 receptor
Th: T helper cell
Treg: Regulatory T cell
