# Supplementary material for: Increased proportions of circulating PD-1+ CD4+ memory T cells and PD-1+ regulatory T cells associate with good response to prednisone in pulmonary sarcoidosis
Source: Respir Res. 2024 May 7;25:196. doi: 10.1186/s12931-024-02833-y (PMC11075187; doi:10.1186/s12931-024-02833-y)
Supplement: Supplementary file 1 — Supplementary Material 1: Supplementary Table 1. Antibodies used for intra- and extracellular staining. [file 12931_2024_2833_MOESM1_ESM.docx]

**Supplemental Table 1: Antibodies used for flow cytometry**

| **Antibody** | **Alternative name** | **Function / expressed on** | **Conjugate** | **Clone** | **Manufacturere** |
| --- | --- | --- | --- | --- | --- |
| CD3 |  |  | APC-Cy™7 | SK7 | BD Biosciences |
| CD4 |  |  | PE-Cy™7 | SK3 | BD Biosciences |
| CD8 |  |  | BB790-P | SK1 | BD Biosciences |
| CD25 | IL-2Rα | Early activation marker | BB515 | 2A3 | BD Biosciences |
| CD27 |  | Activation marker | PE | O323 | BD Biosciences |
| CD28 |  | Costimulation | BV786 | CD28.2 | BD Biosciences |
| CD45RA |  | Naive T-cell | BV480 | 5H9 | BD Biosciences |
| CD45RO |  | Memory T-cell | BV750 | UCHL1 | BD Biosciences |
| CD95 | Fas | Apoptosis marker | APC | DX2 | BD Biosciences |
| CD127 | IL-7Rα |  | PE-CF594 | HIL-7R-M21 | BD Biosciences |
| CD152 | CTLA4 | Co-Inhibitory marker | PE-Cy™5 | BNI3 | BD Biosciences |
| CD279 | PD-1 | Co-Inhibitory marker | BB700 | Eh12.1 | BD Biosciences |
| γδ TCR |  | γδ T-cell | BV421 | 11F2 | BD Biosciences |
