# Supplementary figures and images for: Increased proportions of circulating PD-1+ CD4+ memory T cells and PD-1+ regulatory T cells associate with good response to prednisone in pulmonary sarcoidosis
Source: Respir Res. 2024 May 7;25:196. doi: 10.1186/s12931-024-02833-y (PMC11075187; doi:10.1186/s12931-024-02833-y)

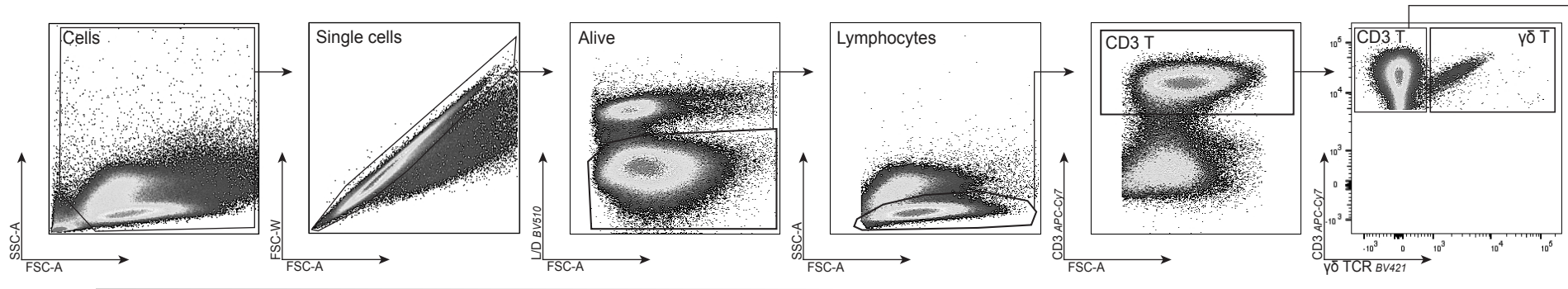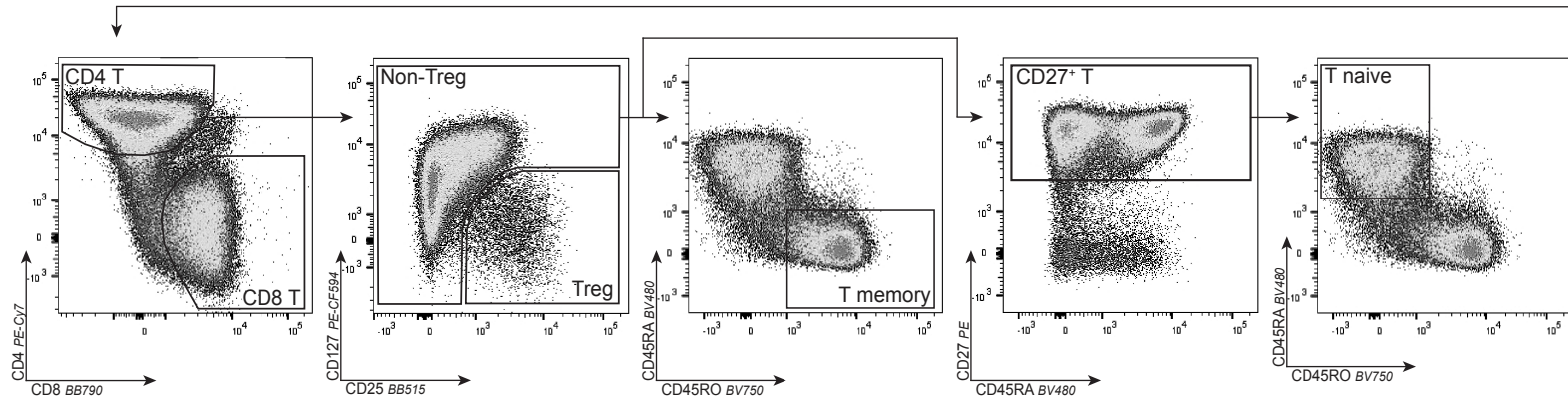

Supplement: Supplementary file 2 — Supplementary Material 2: Suppl. Figure 1. Gating strategy of human T cell subsets. Gating strategy for the indicated human T cell populations in peripheral blood mononuclear cell fractions. Resting T cells: CD127+/-CD25--, Activated T cells: CD127+ CD25+, Tregs: CD127- CD25high. [file 12931_2024_2833_MOESM2_ESM.pdf]

**A**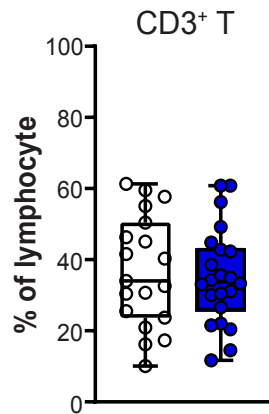**B**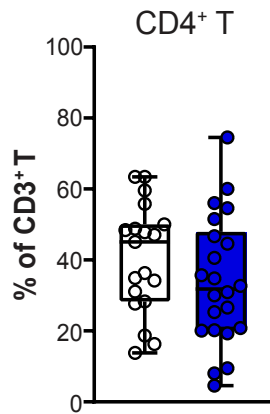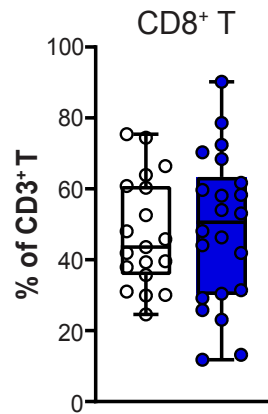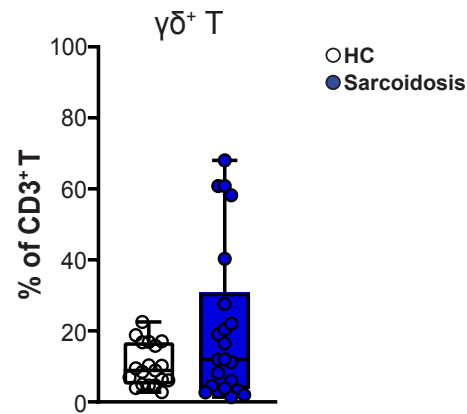**C**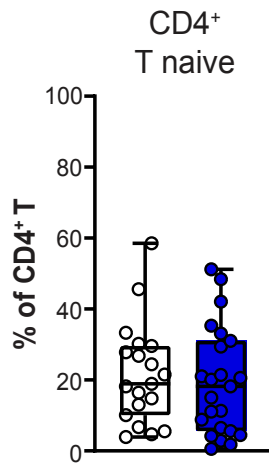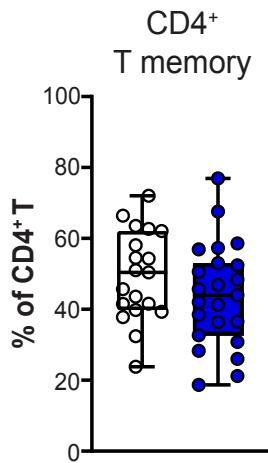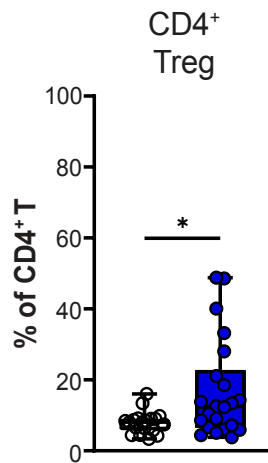

Supplement: Supplementary file 3 — Supplementary Material 3: Suppl. Figure 2. Proportions of T cell subsets in healthy controls and patients with sarcoidosis. (A) Proportions of CD3+ T cells of total lymphocytes in peripheral blood mononuclear cell fractions. (B) Proportions of CD4+, CD8+ and γδ+ T cells of total CD3+ T cells (C) Proportions of CD4+ T naïve, T memory and Treg cells of total CD4+ T cells. Symbols represent individual values in healthy controls (HCs; open circles) and total sarcoidosis patients (Sarcoidosis; blue circles). All data were measured by flow cytometry. Mann-Whitney U test was used to calculate significant differences between two groups. *p < 0.05, **p < 0.01 and ***p < 0.001. [file 12931_2024_2833_MOESM3_ESM.pdf]

**A**

### CD4<sup>+</sup> T memory

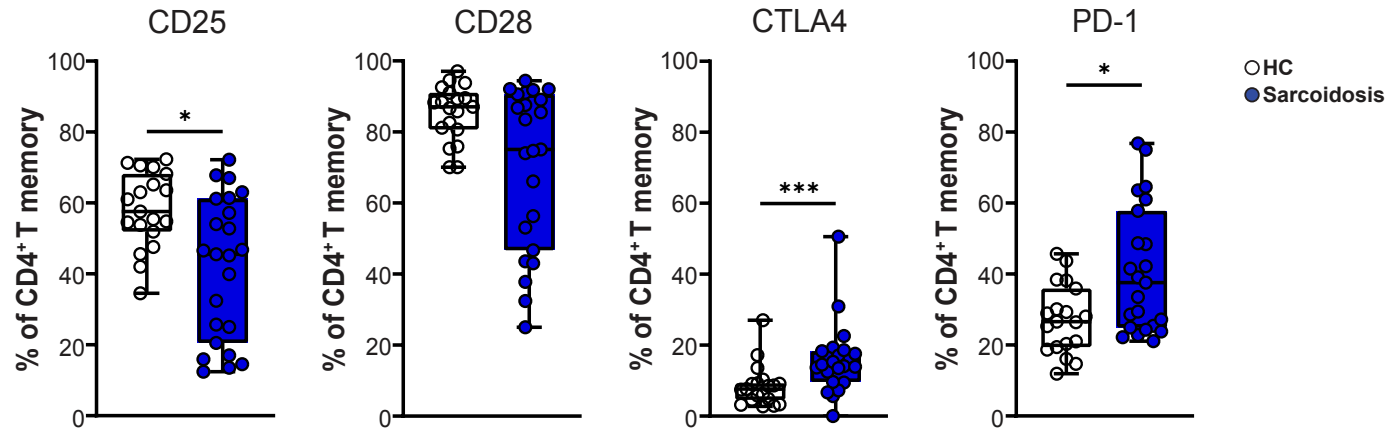**B**

### CD4<sup>+</sup> Treg

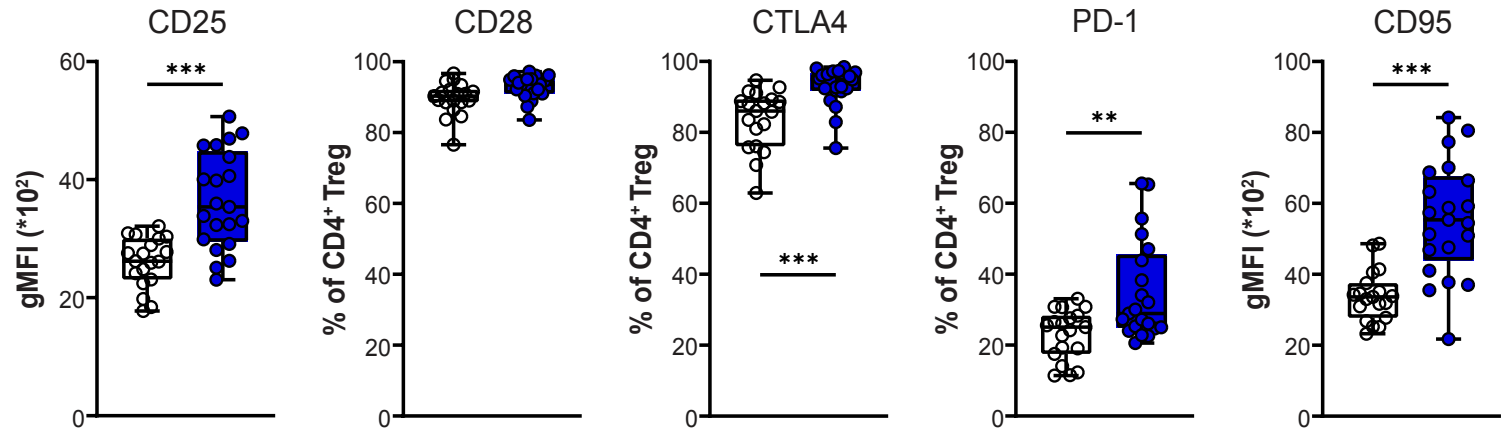

Supplement: Supplementary file 4 — Supplementary Material 4: Suppl. Figure 3. Expression of activation markers on CD4+ memory T cells and Tregs in healthy controls and patients with sarcoidosis. (A) Proportions of CD4+ memory T cells expressing CD25, CD28, CTLA4 and PD-1. (B) Proportions of Tregs expressing CD28, CTLA4 and PD-1. Expression of CD25 and CD95 on Tregs is depicted in gMFI. Symbols represent individual values in healthy controls (HCs; open circles) and total sarcoidosis patients (Sarcoidosis; blue circles). All data were measured by flow cytometry. Mann-Whitney U test was used to calculate significant differences between two groups. *p < 0.05, **p < 0.01 and ***p < 0.001. [file 12931_2024_2833_MOESM4_ESM.pdf]

**A**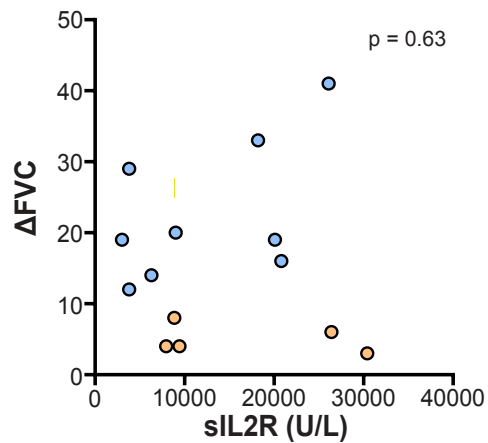**B****CD4<sup>+</sup> T memory**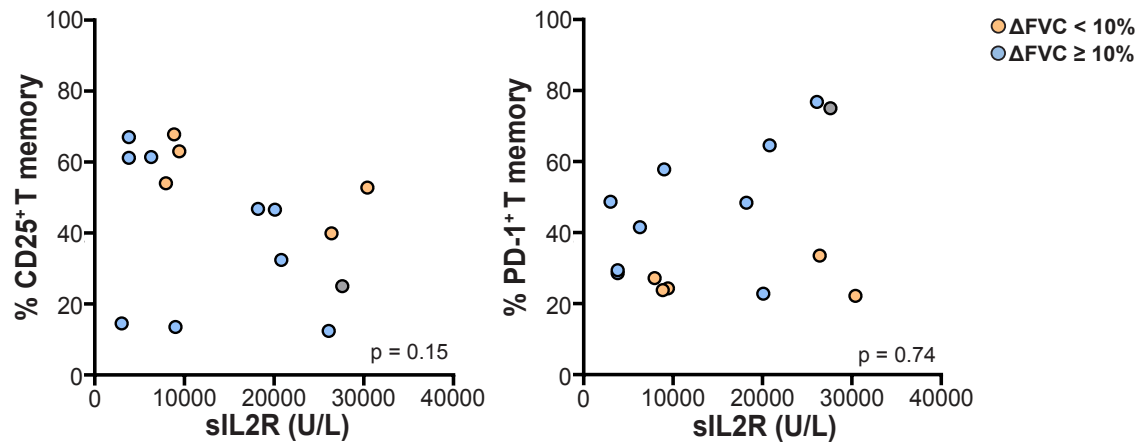**C****CD4<sup>+</sup> Treg**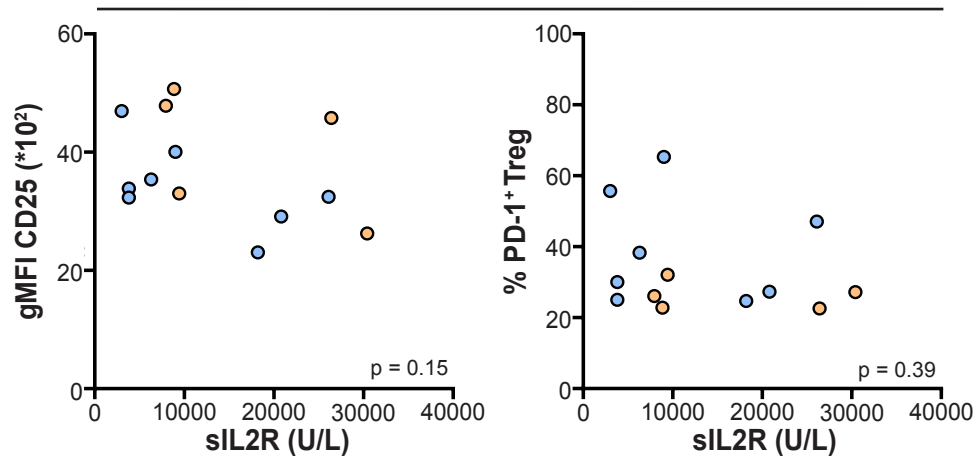

Supplement: Supplementary file 5 — Supplementary Material 5: Suppl. Figure 4. Serum concentration of soluble IL-2 receptor does not correlate with FVC response or T cell activation marker expression. (A) Scatter plots depicting correlation coefficients with p-value between absolute increase in FVC % predicted (∆ FVC) between baseline and 3 months and serum concentration of soluble IL-2 receptor (sIL2R) in U/L in sarcoidosis patients with < 10% and ≥ 10% absolute FVC % predicted increase in 3 months (B) Scatter plot depicting correlation coefficient between proportions of CD4+ memory T cells positive for CD25 (in %; left) and for PD-1 (right) and serum concentration of soluble IL-2 receptor (sIL2R) in U/L in sarcoidosis patients with < 10% and ≥ 10% absolute FVC % predicted increase in 3 months (C) Scatter plot depicting correlation coefficient between baseline expression level of CD25 (in gMFI) (left) or the proportions of Tregs that express PD-1 (right) and serum concentration of soluble IL-2 receptor (sIL2R) in U/L in sarcoidosis patients with < 10% and ≥ 10% absolute FVC % predicted increase in 3 months. Symbols represent individual values in patients with sarcoidosis in the two response groups, as indicated. [file 12931_2024_2833_MOESM5_ESM.pdf]
